# Spectral Methods for Response Enhancement of Microwave Resonant Sensors in Continuous Non-Invasive Blood Glucose Monitoring

**DOI:** 10.3390/bioengineering9040156

**Published:** 2022-04-04

**Authors:** Giovanni Buonanno, Adriana Brancaccio, Sandra Costanzo, Raffaele Solimene

**Affiliations:** 1 DIMES, University of Calabria, 87036 Rende, Italy; giovanni.buonanno@unical.it; 2 Department of Engineering, University of Campania, 81031 Aversa, Italy; adriana.brancaccio@unicampania.it (A.B.); raffaele.solimene@unicampania.it (R.S.); 3 Institute for Electromagnetic Sensing of the Environment (IREA), National Research Council (CNR), 80124 Naples, Italy; 4 National Inter-University Research Center on the Interactions between Electromagnetic Fields and Biosystems (ICEmB), 16145 Genoa, Italy; 5 National Inter-University Consortium for Telecommunications (CNIT), 43124 Parma, Italy; 6 Department of Electrical Engineering, Indian Institute of Technology Madras, Chennai 600036, India

**Keywords:** microwaves resonant biosensors, noninvasive continuous blood glucose monitoring, signal processing

## Abstract

In this paper, the performance of three recent algorithms for the frequency-response enhancement of microwave resonant sensors are compared. The first one, a single-step algorithm, is based on a couple of direct-inverse Fourier transforms, giving a densely sampled response as a result. The second algorithm exploits an iterative procedure to progressively restricts the frequency response. The final one is based on the super-resolution MUSIC algorithm. The comparison is carried out through a Monte Carlo analysis. In particular, synthetic signals are firstly exploited to mimic the frequency response of a resonant microwave sensor. Then, experimental data collected from water-glucose solutions are adopted as validation test for potential applications in noninvasive blood-glucose monitoring.

## 1. Introduction

Diabetes is an extremely widespread disease, potentially leading to very severe implications [1]. In such a context, a number of efforts are being carried out for the development of methodologies and devices enabling a noninvasive continuous glucose detection [2,3,4,5,6].

As is well known, glucose levels are typically measured by taking blood drops from patients’ fingers, and placing them on chemically pretreated strips [7]. To have an accurate control, this operation must be performed several times each day, thus leading to uncomfortable situation for the patients. Moreover, continuous monitoring cannot be assured.

From this perspective, the adoption of microwave sensors [8,9,10,11,12,13,14,15,16,17,18] can provide a valid tool to realize a noninvasive control, one that does not affect the usual life activities of patients. Resonant sensors [19] are particularly attractive for this, as they directly relate the frequency variations of the output response to the electromagnetic changes of the targeted biological tissue (i.e., blood), from which the interested parameter (i.e., glucose concentration) can be subsequently retrieved. However, such sensors may exhibit a low quality factor, and hence low resolution, depending on their specific structure, as well as due to unavoidable losses. Furthermore, when the sampling frequency step is not sufficiently fine, the resonant sensor may fail to capture the true resonant frequency, thus reducing the accuracy of the measurement process.

A useful way to overcome the above issues is to apply signal-processing techniques, able to increase the resolution of the frequency response, as well as the accuracy of the reconstruction process, but avoiding the need for sophisticated and expensive measuring devices or equipments [20,21,22]. In this paper, a signal-processing approach for the response sharpening of microwave resonant sensors is proposed. The idea is to numerically focus on a restricted portion of the sensor curve, in the proximity of the resonance position, by subsequently applying proper signal processing to enhance the resolution in this portion. The outlined approach significantly increases the accuracy of the reconstruction process, but avoids the adoption of a huge number of samples and/or sophisticated hardware otherwise required to achieve the same resolution level. In the paper, three specific techniques are discussed and compared. The first one is based on a single iteration of a couple of direct-inverse Fourier transforms [20]; the second one relies on multiple iterations of these pairs [21]; and, finally, the third algorithm is based on the MUSIC method [22]. A deep analysis of the above three methodologies is conducted, by making a comparison of their performances, and highlighting their respective advantages and weaknesses. The analysis is first carried out in terms of synthetic signals, specifically generated ad hoc. Experimental data measured by a resonant microwave sensor on water-glucose solutions are also considered as validation test.

## 2. Algorithms Description

In this section, the algorithms under comparison are briefly described, by reporting all essential elements to make the paper self-consistent.

**Figure 1:**
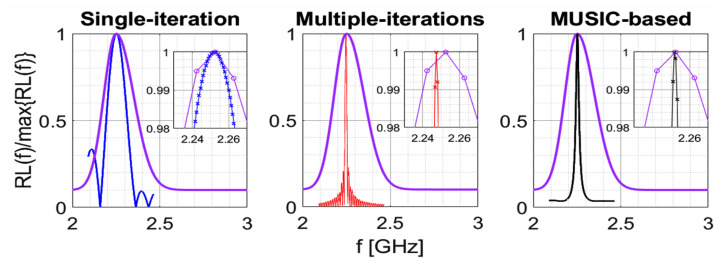
Application example of the three sharpening algorithms under analysis.

Let us consider the purple curve shown in Figure 1, which represents a typical return loss function, RL(f), related to a microwave sensor [23], namely:

(1) $$RL\left(f\right)=-20\;log_{10}\left|\Gamma \left(f\right)\right|$$

In particular, the function Γ(f) represents the reflection coefficient measured at the sensor input, within the observation band $\left[f_{min},f_{MAX}\right]$. As can be seen, the signal has a significant dispersion around its maximum value, due both to the losses as well as to the specific sensor configuration. Therefore, its response is “far” from being an ideal Dirac impulse at the resonance frequency. It must be taken into account that, if the acquisition frequency step is not sufficiently fine, the return loss sample associated to the resonant frequency may not be captured. Moreover, the relatively small shifts in the resonant frequency may not be correctly identified. However, as will be shown in the following, the three considered algorithms successfully cope with the above issue.

In order to highlight the key points of these algorithms, we can assume, without loss of generality, that the return loss function Γ(f) satisfies the condition $RL\left(f_{min}\right)<RL\left(f_{MAX}\right)$. Furthermore, it must be noted that acting on the return loss by means of Fourier transform means operating with the *complex cepstrum* [24] of the inverse Fourier transform of the magnitude of the reflection coefficient.

### 2.1. Data Preprocessing

The three algorithms we are going to compare share the same initial preprocessing steps on the acquired return loss. The common operations are described in the following:

1 Making *M* return loss acquisitions and performing the sample average of them. The value of *M* must be set to obtain a sufficiently smooth signal as that of Figure 1. The aim of this step is to realize a noise filter, without affecting the “true” signal.
2 Determining the frequency value $\tilde{f}$, such that $RL\left(\tilde{f}\right)=RL\left(f_{MAX}\right)$.
3 Defining:
  (2) $$\tilde{RL}\left(f\right)=\left\{\begin{array}{cc} RL(\tilde{f}) & f_{min}\leq f\leq \tilde{f}\\ RL(f), & \tilde{f}\leq f\leq f_{MAX} \end{array}\right.$$
4 Defining the “new” version of the return loss as $\bar{RL}\left(f\right)=\tilde{RL}\left(f\right)-RL\left(f_{MAX}\right)$, which is devoid of endpoint contributions.
5 Setting the real parameter 0<α<1.
6 Identifying the two values ${\tilde{f}}_{min}>f_{min}$ and ${\tilde{f}}_{MAX}<f_{MAX}$ such that:
  (3) $$\frac{RL({\tilde{f}}_{min})}{max\{RL(f)\}}=\frac{RL({\tilde{f}}_{MAX})}{max\{RL(f)\}}=\alpha$$
7 Computing the inverse Fourier transform of the portion of $|\bar{RL}\left(f\right)|$ enclosed in the interval $\left[f_{min},f_{MAX}\right]$ (for $t\in [t_{min},t_{MAX}]$), namely:
  (4) $$rl\left(t\right)=\int_{f_{min}}^{f_{MAX}}\left|\bar{RL}\left(f\right)\right|\;e^{j2\pi \;t\;f}\;df$$
  for which the *unwrapped* version of the phase ϕ(t)=∠rl(t) is almost linear in the main-lobe region of |rl(t)|, where the information about the resonance frequency is actually encoded. The magnitude of $\bar{RL}\left(f\right)$ is considered to cut any negative values with small magnitudes, which may exist due to noise.
8 Setting the real parameter 0<β<1.
9 Identifying the two values ${\tilde{t}}_{min}$ and ${\tilde{t}}_{MAX}$ such that:
  (5) $$\frac{\left|rl({\tilde{t}}_{min})\right|}{max\{|rl(t)|\}}=\frac{\left|rl({\tilde{t}}_{MAX})\right|}{max\{|rl(t)|\}}=\beta$$

### 2.2. Single-Step Algorithm

This algorithm is based on a single iteration of a pair of Fourier transformations. Actually, to make a comparison based on the same settings, we consider here a slightly modified version of the algorithm presented in [20]. In particular, the end points are eliminated in [20] after determining the extremes ${\tilde{f}}_{min}$ and ${\tilde{f}}_{MAX}$, while in this work the end points are first removed, and then the above extremes are identified. Assuming the pre-processing steps 1 to 9 have already been carried out, this algorithm requires the following additional steps (please note the progressive numbering starting from 10):

10 Computing the new version of the return loss (for $f\in [{\tilde{f}}_{min},{\tilde{f}}_{MAX}]$), namely:
  (6) $$\hat{RL}\left(f\right)=\left|\int_{{\tilde{t}}_{min}}^{{\tilde{t}}_{MAX}}e^{j\phi (t)}\;e^{-j2\pi \;t\;f}\;dt\right|$$
  which is narrower with respect to the original one.
11 Assuming the point of maximum of $\hat{RL}\left(f\right)$ as the estimate of the resonance frequency, $f_{est}$.

Before discussing the other two sharpening methods, it is useful to make some clarifications regarding the numerical implementation. Indeed, from a practical point of view, we operate with digital signals, because the algorithm starts with the acquisition of $N_{f}$ samples of the return loss within the band $\left[f_{min},f_{MAX}\right]$. Accordingly, $\tilde{RL}\left(f\right)$, $\bar{RL}\left(f\right)$ and $\hat{RL}\left(f\right)$ are discrete-frequency signals. More precisely, $\tilde{RL}\left(f\right)$ and $\bar{RL}\left(f\right)$ are sampled in $N_{f}$ points of the domain $\left[f_{min},f_{MAX}\right]$; instead, $\hat{RL}\left(f\right)$ is sampled in ${\hat{N}}_{f}$ ($>>N_{f}$) points of domain $\left[{\tilde{f}}_{min},{\tilde{f}}_{MAX}\right]$. Hence, assuming uniform sampling, the initial frequency step $\Delta f=\left(f_{MAX}-f_{min}\right)/\left(N_{f}-1\right)$ is much larger than the final one $\hat{\Delta }f=\left({\tilde{f}}_{MAX}-{\tilde{f}}_{min}\right)/\left({\hat{N}}_{f}-1\right)$. From the foregoing, it also follows that the values ${\tilde{f}}_{min}$ and ${\tilde{f}}_{MAX}$ approximately satisfy the equality relative to the normalised return loss in point 3. The same argumentation applies to $\tilde{f}$ in point 4.

Obviously, even in the time domain we operate with digital signals. Taking into account that the time window [−0.5/Δf,0.5/Δf] corresponds to the period of the inverse discrete-time Fourier transform of a uniformly sampled frequency signal with step equal to Δf, the signal rl(t) is sampled in $N_{t}$ points of the interval $\left[t_{min},t_{MAX}\right]$, with $t_{min}=-t_{MAX}=-0.5/\Delta f$, where $N_{t}$ is independent of $N_{f}$ and ${\hat{N}}_{f}$.

Finally, Equation (4) is implemented as the sampled version of the inverse discrete-time Fourier transform of $|\bar{RL}\left(f\right)|$, while Equation (6) is implemented as the magnitude of the sampled version of the discrete-time Fourier transform of the term $e^{j\phi (t)}$. In general, discrete-time Fourier transformations are used in order to have greater flexibility in terms of sampling, in the sense that even nonuniform sampling is very easy to implement.

### 2.3. Iterative Algorithm

The iterative algorithm is based on an “uncertain” (i.e., not a priori known ) number of iterations of the pair of Fourier transformations and, actually, it shares points 1 to 10 with the previous algorithm. Assuming that the above preprocessing steps have already been carried out, the current algorithm consists of the following additional steps (please observe that also in this case the progressive numbering starts from 10):

10 Computing the new version of the return loss (for $f\in [{\tilde{f}}_{min},{\tilde{f}}_{MAX}]$), namely:
  (7) $$\hat{RL}\left(f\right)=\left|\int_{{\tilde{t}}_{min}}^{{\tilde{t}}_{MAX}}e^{j\phi (t)}\;e^{-j2\pi \;t\;f}\;dt\right|$$
  which is narrower with respect to the original one.
11 Computing the new version of rl(t), namely:
  (8) $$rl\left(t\right)=\int_{{\tilde{f}}_{min}}^{{\tilde{f}}_{MAX}}{\hat{RL}}^{2}\left(f\right)\;e^{j2\pi t\;f}\;df$$
  which presents a wider magnitude main-lobe, so that its unwrapped phase is almost linear over a longer time interval. Again, Equation (8) is implemented as the sampled version of the inverse discrete-time Fourier transform of the function ${\hat{RL}}^{2}\left(f\right)$.
12 Repeating the pre-processing step 9 and steps 10 and 11 (of the current algorithm) as long as the condition $\left|rl\left(t\right)\right|/\max\left\{|rl\left(t\right)|\right\}\geq \beta \;\;\forall \;\;t\in \left[t_{min},t_{MAX}\right]$ is not fulfilled, and finally computing the final return loss by Equation (6), whose point of maximum gives the estimated resonance frequency, $f_{est}$.

For the above algorithm, the same implementation considerations of the previous procedure also apply.

### 2.4. MUSIC-Based Algorithm

MUSIC-based algorithm consists in determining the MUSIC pseudospectrum [25,26], starting from the phase of the return-loss function rl(t) provided by Equation (4). As stated above, it shares some points with the other two mentioned procedures. In order to make the comparison based on the same identical parameters, we consider here a slightly modified version of the MUSIC-based algorithm with respect to that presented in [22], thus also considering the parameter β. Assuming the preprocessing steps 1 to 9 have already been carried out, the current algorithm consists of the following additional steps (please, observe again that the numbering starts at 10, in continuity with the common preliminary 1–9 steps described in Section 2.1):

10 Computing the so-called (${\tilde{N}}_{t}\times {\tilde{N}}_{t}$) *correlation matrix* [26], namely:
  (9) $$\underline{\underline{R}}=\underline{\tilde{rl}}\;{\underline{\tilde{rl}}}^{H}$$
  in which:
  (10) $$\underline{\tilde{rl}}={\left(e^{j\phi ({\tilde{t}}_{min})},e^{j\phi ({\tilde{t}}_{min}+\Delta t)},...,e^{j\phi ({\tilde{t}}_{MAX})}\right)}^{T}$$
  where ${\tilde{N}}_{t}$ is the number of samples of the function $e^{j\phi (t)}$ falling within the interval $\left[{\tilde{t}}_{min},{\tilde{t}}_{MAX}\right]$, $\Delta t=\left({\tilde{t}}_{MAX}-{\tilde{t}}_{min}\right)/\left({\tilde{N}}_{t}-1\right)$, the superscript *H* stands for transposition and conjugation, and the superscript *T* stands for transposition. It is worth noting that no additional decorrelation procedure [26] (between spectral lines to be estimated) is needed here, as there is only one resonance frequency (spectral line).
11 Performing the eigendecomposition of $\underline{\underline{R}}$ as:
  (11) $$\underline{\underline{R}}=\underline{\underline{V}}\;\underline{\underline{\Lambda }}\;{\underline{\underline{V}}}^{-1}$$
  in which $\underline{\underline{V}}$ and $\underline{\underline{\Lambda }}$ are the eigenvectors and eigenvalues matrices, respectively.
12 Computing the MUSIC eigenspectrum (for $f\in [{\tilde{f}}_{min},{\tilde{f}}_{MAX}]$) [27] as:
  (12) $$P\left(f\right)=\frac{{\underline{a}}^{H}\left(f\right)\;\underline{a}\left(f\right)}{{\underline{a}}^{H}\left(f\right)\;\tilde{\underline{\underline{V}}}\;{\tilde{\underline{\underline{V}}}}^{H}\;\underline{a}\left(f\right)}$$
  in which $\tilde{\underline{\underline{V}}}$ consists of the columns of $\underline{\underline{V}}$ corresponding to the smallest ${\tilde{N}}_{t}-1$ eigenvalues contained in $\underline{\underline{\Lambda }}$, and
  (13) $$\underline{a}\left(f\right)={\left(e^{j2\pi f{\tilde{t}}_{min}},e^{j2\pi f({\tilde{t}}_{min}+\Delta t)},...,e^{j2\pi f{\tilde{t}}_{MAX}}\right)}^{T}$$
  is the so-called *steering vector*. As for $\hat{RL}\left(f\right)$, the eigenspectrum is also sampled at ${\hat{N}}_{f}$ points belonging to the interval $\left[{\tilde{f}}_{min},{\tilde{f}}_{MAX}\right]$.
13 Assuming as final return loss the eigenspectrum given by Equation (12) and, consequently, the point of maximum of this function as the estimate of the resonance frequency $f_{est}$. Note that this assumption is consistent with the fact that the MUSIC method is essentially a spectral estimation method.

Figure 1 shows an example of the application of the three described algorithms to the return loss depicted with purple line, which is given by the following expression (in the frequency band $\left[f_{min},f_{MAX}\right]=\left[2,3\right]$ GHz):

(14) $$RL\left(f\right)=\left\{\begin{array}{cc} 18\;e^{-\frac{{\left(f-f_{true}\right)}^{2}}{2\;{\left(0.07\;10^{9}\right)}^{2}}}+2, & 2\;GHz\leq f\leq f_{true}\\ 18\;e^{-\frac{{\left(f-f_{true}\right)}^{2}}{2\;{\left(0.10\;10^{9}\right)}^{2}}}+2, & f_{true}\leq f\leq 3\;GHz \end{array}\right.$$

in which $f_{true}$ is the point of maximum, equal to 2.25 GHz in the current example. The number of samples on the purple curve is equal to $N_{f}=101$. The three algorithms have been implemented by using α=0.1, β=0.05, $N_{t}={\hat{N}}_{f}=512$. It can be appreciated that all algorithms provide a tighter final return loss, as compared to the initial one. In particular, taking advantage from the additional steps, the iterative and the MUSIC-based algorithms provide an even tighter final return loss than that given by the single-step algorithm. However, it is even more important to compare the methods in terms of accuracy and precision in the estimation of the resonant frequency. This specific aspect will be discussed in the following section.

## 3. Methods Comparison

The following percentage error is chosen as a metric to compare the three sharpening algorithms described in the previous section:

(15) $$PE\left(\alpha ,\beta ,N_{f},N_{t},{\hat{N}}_{f},f_{true}\right)=\left|\frac{f_{true}-f_{est}}{f_{true}}\right|\times 100\%$$

in which $f_{true}$ denotes the true value of the resonance frequency. As it can be seen, the dependence of the percentage error on the different parameters involved in the various algorithms and the true value of the resonance frequency has been explicitly stated. Then, in the next section, the three algorithms are compared with respect to the values assumed by such parameters. We specify that all the involved functions (i.e., RL(f), $\bar{RL}\left(f\right)$, $\tilde{RL}\left(f\right)$, $\hat{RL}\left(f\right)$, rl(t)) are uniformly sampled.

A Monte Carlo analysis is performed, where the parameters affecting expression (15) are generated iteratively as uniform random variables. At each iteration, the percentage error is determined by means of Equation (15), so that its cumulative distribution function can be determined. Furthermore, the ties between PE and each of the six parameters are also investigated by resorting to scatter plots.

The assessment described above refers to the case of synthetic signals, for which the true resonance frequency is exactly known. When considering experimental contexts, such a true value is not known at all, and so the algorithms under consideration perform a blind resonance frequency estimation. In this case, the percentage error cannot be computed, so that we perform the statistical characterisation of the estimated resonant frequency as a function of the quintuple $\left(\alpha ,\beta ,N_{f},N_{t},{\hat{N}}_{f}\right)$.

## 4. Numerical Results

As anticipated, this section presents the results of a stochastic assessment with respect to signals generated ad hoc; subsequently, the dispersion of the resonance frequency estimation with respect to experimental data (i.e., when the true resonance frequency value is not known) is evaluated.

Let us start with the results related to synthetic signals. For this case, the continuous parameters are generated as uniform random variables, while the discrete ones are uniform multinomial random variables, within the following respective intervals: α∈[0.1,0.9], β∈[0.05,0.45], $N_{f}\in \left[100,900\right]$, $N_{t}\in \left[512,4608\right]$, ${\hat{N}}_{f}\in \left[512,4608\right]$, $f_{true}\in \left[2.25,2.75\right]$ GHz. For each subcase, 500 generations/computations are carried out. The synthetic signals are generated by means of Equation (14) within the (initial) observation band $\left[f_{min},f_{MAX}\right]=\left[2,3\right]$ GHz. Since here $f_{true}$ is a random variable, it follows that RL(f) is a stochastic process, for which each synthetic generated signal is a realisation (sample path).

**Figure 2:**
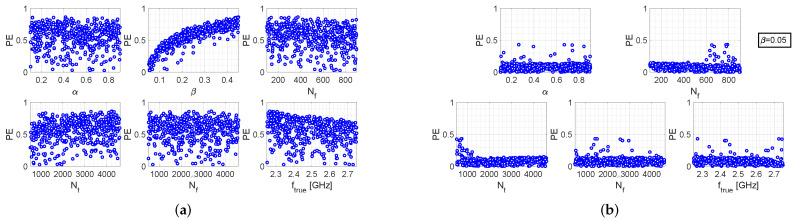
Performance evaluation of the single-step algorithm: (**a**) Scatter plots between PE and the various setting parameters, when the latter are all uniform random variables (**b**) Scatter plots between PE and the various setting parameters, when β=0.05 and all other parameters are uniform random variables.

Figure 2a refers to the single-step algorithm and shows six scatter plots, each depicting the percentage error versus one of the parameters, when all other parameters assume uniformly distributed random values. As can be readily noticed, a certain linear relationship exists between PE and β, whereas it does not appear any particular dependence on the other parameters. It can just be observed that for higher values of $f_{true}$ the maximum value of PE decreases almost linearly, which is reasonable as PE is defined. However, such a dependence is not as pronounced as the one between PE and β.

The above considerations are also supported by the scatter plots shown in Figure 2b, referring to the case in which all the parameters are still randomly generated, except for parameter β which is fixed at the value 0.05. In this case, a clear decrease of the percentage error is observed. As a matter of fact, in the previous case PE reaches values equal to 0.9%, with the majority of observations concentrated approximately between 0.4% and 0.8%. Instead, in the fixed-β case, it can be observed that most of the observations are concentrated below 0.2%, and only few points lie between 0.2% and 0.5%.

**Figure 3:**
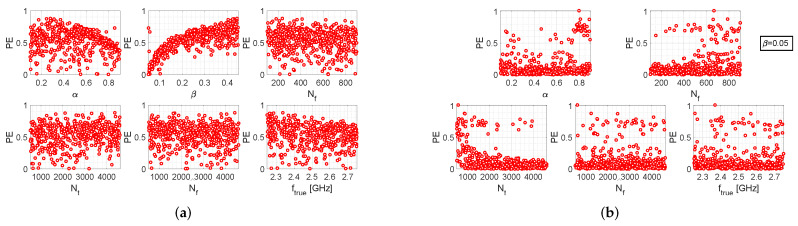
Performance evaluation of the iterative algorithm: (**a**) scatter plots between PE and the various setting parameters, when the latter are all uniform random variables; (**b**) scatter plots between PE and the various setting parameters, when β=0.05 and all other parameters are uniform random variables.

**Figure 4:**
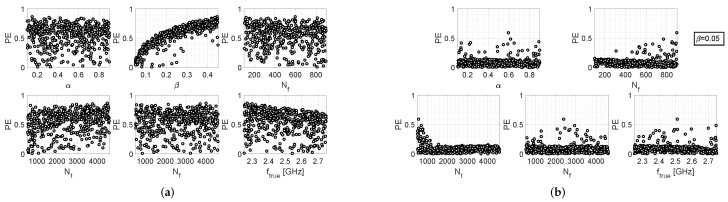
Performance evaluation of the MUSIC-based algorithm: (**a**) scatter plots between PE and the various setting parameters, when the latter are all uniform random variables; (**b**) scatter plots between PE and the various setting parameters, when β=0.05 and all other parameters are uniform random variables.

Similar arguments can be made for the results provided by the other two algorithms, which are reported in Figure 3 and Figure 4. However, it should also be noted that the iterative algorithm gives a more modest improvement in the percentage error levels in the fixed-β case (Figure 3b), as compared to the other two algorithms. In any case, it can be observed that, for the fixed-β case, higher values of $N_{t}$ are more convenient for all three algorithms, since higher values of PE are associated with lower values of $N_{t}$; in the other way, which turns out to be counter-intuitive, choosing lower values of $N_{f}$ (i.e., the number of samples of the return loss acquired by the measurement instrumentation) increases the probability of cutting out higher values of PE. In conclusion, by comparing the scatter plots of Figure 2b, Figure 3b and Figure 4b, it can be stated that the single-step algorithm is the best-performing one in terms of estimation accuracy of the resonance frequency, since the maximum value of PE is lower than that achieved with the two other algorithms.

**Figure 5:**
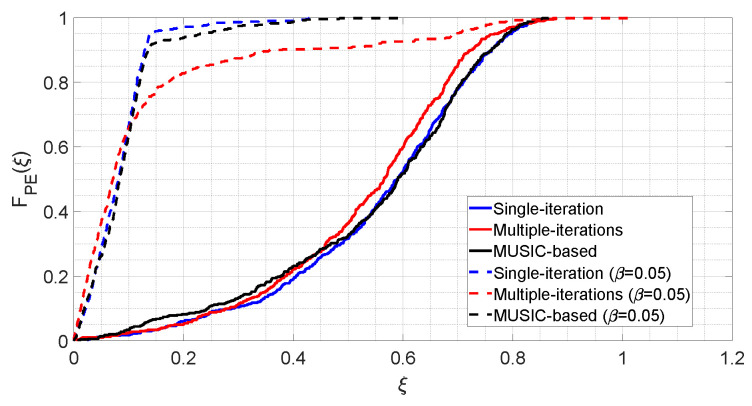
Cumulative distribution functions of the percentage error related to the three sharpening algorithms.

The results observed in Figure 2, Figure 3 and Figure 4 are condensed in Figure 5, which shows the distributions of PE for the six considered cases.

Solid lines in Figure 5 represent the case in which all parameters are random variables, summarizing what is visible in Figure 2a, Figure 3a and Figure 4a. It can be seen that, roughly for all three algorithms, the value ξ=0.2 represents the 5th percentile, whereas the value ξ=0.8 is the 95th percentile. The median is around ξ=0.6.

Dashed lines in Figure 5 represent the fixed-β cases, summarizing what is seen in Figure 2b, Figure 3b and Figure 4b. As it can be appreciated, these lines are shifted to the left with respect to the solid ones, which indicates that parameter PE assumes lower values, and therefore more accurate estimates are provided. Furthermore, the dashed lines distributions also show a faster growth rate than the solid ones, which therefore means less dispersion of the observations, and therefore higher precision. As a matter of fact, in the fixed-β case the 5th percentile is obtained at ξ<0.05 for all three algorithms. Regarding the 95th percentile, with the single-step and the MUSIC-based algorithms, it stays around ξ=0.2, whereas with the iterative algorithm it is close to ξ=0.7, which is coherent with the result shown in Figure 3b.

## 5. Experimental Results

**Figure 6:**
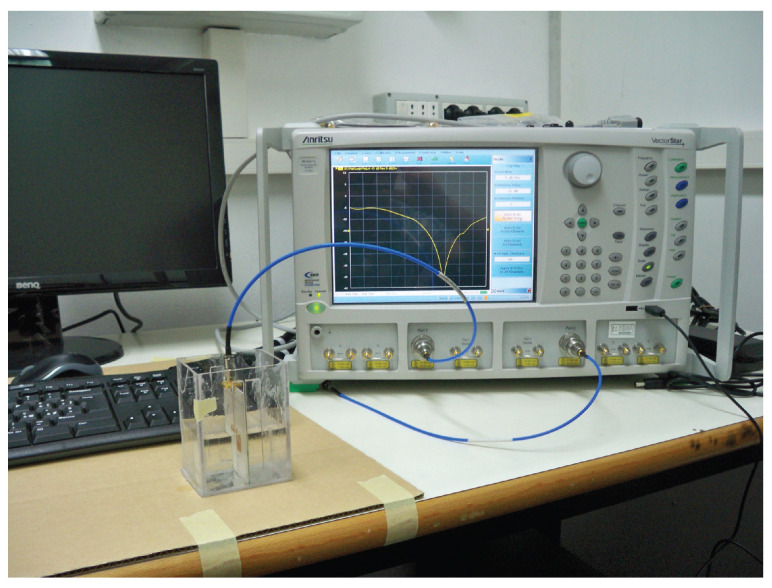
Measurement setup into ERMIAS Lab at University of Calabria [10].

In the present Section, the evaluation of results coming from the analysis of experimental data is carried out. The measurement setup is illustrated in Figure 6, where the microwave-resonant sensor is immersed into a water-glucose solution, and connected to the Vector Network Analyzer (VNA) Anritsu VectorStar, in order to acquire the return loss (amplitude of the reflection scattering parameter). This represents the output response of the sensor, clearly exhibiting a pronounced resonance at a specific frequency within the Industrial, Scientific, Medical (ISM) band. The sensor configuration, described in [10], is represented by a standard inset-fed resonant microstrip patch antenna, printed on a thin (0.762 mm height) dielectric substrate, having a high permittivity ($\epsilon_{r}=10$), in order to reduce the effect of the environmental properties as much as possible [10]. The sensor design is optimised, in terms of its resonant response, by means of the Ansys software, after a preliminary dielectric characterization of water-glucose solutions, from which an accurate dielectric model for the blood is derived [17]. In particular, an optimum matching condition (i.e., resonant condition) for the microwave sensor is properly achieved at the frequency $f_{0}=2.4$ GHz.

**Figure 7:**
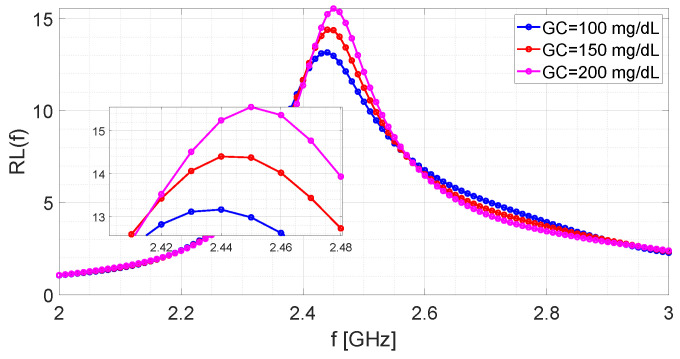
Return loss measured by the microwave-resonant sensor for different glucose concentrations.

Figure 7 shows the return losses provided by the microwave sensor in correspondence to three different values of the glucose concentration (GC). The number of samples, equispaced in the band [2,3] GHz, is equal to $N_{f}=101$. Consequently, the frequency sampling step is fixed to $\Delta_{f}=10$ MHz.

**Figure 8:**
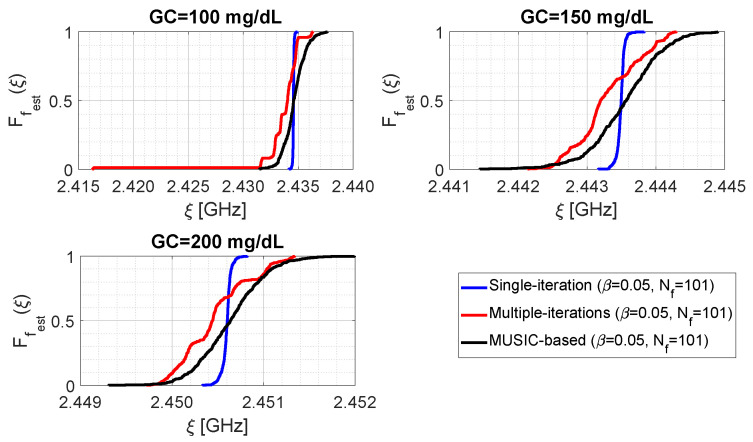
Cumulative distribution functions of the resonant frequency estimate for the three sharpening algorithms, as a function of the glucose concentration.

Figure 8 shows the distributions of parameter $f_{est}$ for the three examined algorithms at different glucose concentrations. The distributions are obtained under the settings $N_{f}=101$, β=0.05, and generating the other parameters as random uniform variables in the ranges α∈[0.1,0.9], $N_{t}\in \left[512,4608\right]$ and ${\hat{N}}_{f}\in \left[512,4608\right]$. As it can be seen, the single-step algorithm provides less dispersed data, and is therefore equipped with greater precision, since the blue lines are more “vertical” than the others. Furthermore, it can be observed that for GC=100 mg/dL the distribution related to the iterative algorithm (red line) shows a long left tail, due to the presence of some outliers around 2.4163 GHz. By looking at Table 1, it can be recognised that although MUSIC-based algorithm presents almost the same dispersion as the multiple-iteration one (for example, in terms of difference between the 5th and 95th percentiles), both procedures give a value of 0.5 approximately in correspondence of the same value of ξ of the single-step algorithm. Accordingly, the single-step and the MUSIC-based algorithms provide approximately the same median for parameter $f_{est}$; the same situation does not hold for the iterative algorithm. Furthermore, another interesting consideration is that the sample mean and the sample median are almost coincident for each individual case (e.g., referring to Table 1, for GC=100 mg/dL the single-step algorithm gives μ=M=2.4346 GHz). By comparing data shown in Figure 7 with those of Figure 8 and Table 1, it can be intuitively concluded that parameter μ provides a good estimate of the resonance frequency, and the difference $P_{95\%}-P_{5\%}$ is definitely lower than the initial sampling step $\Delta_{f}=10$ MHz. Finally, for the assumed cases, no distribution associated with a given GC value overlaps with another one associated with a different GC (for example, the black curve related to GC=100 mg/dL reaches its maximum value before ξ=2.4400 GHz, while the black curve related to GC=150 mg/dL presents values greater than zero after ξ=2.4410 GHz).

A final consideration must be taken into account in relation to the computational cost. The single-step and the MUSIC-based algorithms involve roughly comparable running times, while the iterative algorithm requires a longer running time, as it can be easily guessed.

## 6. Conclusions

In this work, three different algorithms have been analyzed and compared to increase the accuracy of microwave-resonant sensors for potential applications in the reconstruction of blood glucose concentrations. The first two algorithms are based on pairs of direct-inverse Fourier transformations. In particular, the first one just adopts a single pair, while the second one consists of multiple iterations of this. In particular, the third algorithm is mainly based on a super-resolution spectral estimation method, i.e., the MUSIC algorithm. The comparison of the above procedures has been performed using Monte Carlo analysis, and treating the various involved optimisation parameters as random variables. Firstly, the analysis has been carried out by using synthetic data; then, experimental data obtained by a ISM-resonant microwave sensor from water-glucose solutions have been also considered. From the achieved results, all methods have revealed satisfactory performances in terms of accuracy and precision in the estimation of the resonant frequency, and thus in the reconstruction of the blood glucose concentration. More specifically, the single-step algorithm is revealed to provide the best performance. Furthermore, a major result emerging in the work is related to the possibility to obtain a very accurate estimation of the resonant frequency, even when considering a limited number of acquired samples, thus avoiding the need for sophisticated and expensive measuring hardware.

## Figures and Tables

**Table 1:** Statistics of $f_{est}$ for three different values of the glucose concentration (GC). Symbols μ, σ, M, $P_{5\%}$ and $P_{95\%}$ denotes sample mean, standard deviation, median, 5th percentile, 95th percentile of $f_{est}$, respectively. The parameters are: β=0.05, $N_{f}=101$; α∈[0.1,0.9], $N_{t}=\left[512,4608\right]$ and ${\hat{N}}_{f}\in \left[512,4608\right]$ are uniform random variables. The values of GC are in mg/dL and those of the statistics are in GHz.

	Single-Step				Iterative				MUSIC-Based			
GC	μ	M	$P_{5\%}$	$P_{95\%}$	μ	M	$P_{5\%}$	$P_{95\%}$	μ	M	$P_{5\%}$	$P_{95\%}$
100	2.4346	2.4346	2.4345	2.4347	2.4336	2.4340	2.4316	2.4350	2.4346	2.4346	2.4332	2.4364
150	2.4435	2.4435	2.4434	2.4436	2.4433	2.4432	2.4426	2.4441	2.4435	2.4436	2.4427	2.4444
200	2.4506	2.4506	2.4505	2.4507	2.4505	2.4504	2.4499	2.4511	2.4506	2.4506	2.4501	2.4512

## Data Availability

The data presented in this study are available on request from the corresponding author.

## Abbreviations

The following abbreviations are used in this manuscript:

MUSIC	MUltiple Signal Classification
*PE*	Percentage Error
*GC*	Glucose Concentration
